# Assessment of the evolution of end-tidal carbon dioxide within chest compression pauses to detect restoration of spontaneous circulation

**DOI:** 10.1371/journal.pone.0251511

**Published:** 2021-05-18

**Authors:** Jose Julio Gutiérrez, Mikel Leturiondo, Sofía Ruiz de Gauna, Jesus María Ruiz, Izaskun Azcarate, Digna María González-Otero, Juan Francisco Urtusagasti, James Knox Russell, Mohamud Ramzan Daya

**Affiliations:** 1 Department of Communications Engineering, University of the Basque Country, UPV/EHU, Bilbao, Spain; 2 Bexen Cardio, Ermua, Spain; 3 Emergentziak-Osakidetza, Basque Country Health System, Basque Country, Spain; 4 Oregon Health & Science University (OHSU), Portland, Oregon, United States of America; Fondazione IRCCS Policlinico San Matteo, ITALY

## Abstract

**Background:**

Measurement of end-tidal CO_2_ (ETCO_2_) can help to monitor circulation during cardiopulmonary resuscitation (CPR). However, early detection of restoration of spontaneous circulation (ROSC) during CPR using waveform capnography remains a challenge. The aim of the study was to investigate if the assessment of ETCO_2_ variation during chest compression pauses could allow for ROSC detection. We hypothesized that a decay in ETCO_2_ during a compression pause indicates no ROSC while a constant or increasing ETCO2 indicates ROSC.

**Methods:**

We conducted a retrospective analysis of adult out-of-hospital cardiac arrest (OHCA) episodes treated by the advanced life support (ALS). Continuous chest compressions and ventilations were provided manually. Segments of capnography signal during pauses in chest compressions were selected, including at least three ventilations and with durations less than 20 s. Segments were classified as ROSC or non-ROSC according to case chart annotation and examination of the ECG and transthoracic impedance signals. The percentage variation of ETCO_2_ between consecutive ventilations was computed and its average value, Δ*ET*_avg_, was used as a single feature to discriminate between ROSC and non-ROSC segments.

**Results:**

A total of 384 segments (130 ROSC, 254 non-ROSC) from 205 OHCA patients (30.7% female, median age 66) were analyzed. Median (IQR) duration was 16.3 (12.9,18.1) s. Δ*ET*_avg_ was 0.0 (-0.7, 0.9)% for ROSC segments and -11.0 (-14.1, -8.0)% for non-ROSC segments (*p* < 0.0001). Best performance for ROSC detection yielded a sensitivity of 95.4% (95% CI: 90.1%, 98.1%) and a specificity of 94.9% (91.4%, 97.1%) for all ventilations in the segment. For the first 2 ventilations, duration was 7.7 (6.0, 10.2) s, and sensitivity and specificity were 90.0% (83.5%, 94.2%) and 89.4 (84.9%, 92.6%), respectively. Our method allowed for ROSC detection during the first compression pause in 95.4% of the patients.

**Conclusion:**

Average percent variation of ETCO_2_ during pauses in chest compressions allowed for ROSC discrimination. This metric could help confirm ROSC during compression pauses in ALS settings.

## Introduction

Waveform capnography provides a continuous non-invasive measure of the concentration of partial pressure of carbon dioxide (PCO_2_) during the breathing cycle. The value of PCO_2_ at the end of expiration is known as end-tidal carbon dioxide (ETCO_2_), and can be used to indirectly monitor cardiac output and pulmonary blood flow [1–4].

According to current advanced life support (ALS) guidelines [5, 6], monitoring ETCO_2_ level during resuscitation is beneficial for several reasons including: supervision of cardiopulmonary resuscitation (CPR) quality [7–10], prediction of patient’s outcome [11–15], and early recognition of return of spontaneous circulation (ROSC) [16–19]. The relationship between ETCO_2_ and defibrillation effectiveness could also be useful to guide defibrillation during CPR [20].

Early recognition of ROSC prevents unnecessary administration of chest compressions and adrenaline [21, 22]. However, inappropriate interruptions of chest compressions can reduce the chance of survival [23–25]. In order to provide best chance of survival, interruptions in CPR for assessing the presence of ROSC should be minimized [5, 6].

Accurate detection of ROSC using waveform capnography in pre-hospital ALS settings remains a challenge. Existing methods are based on the detection of sudden increases in the level of ETCO_2_ [16–19]. However, the obtained results generally lack sensitivity or specificity. Quality of CPR influences the interpretation of ETCO_2_ [21, 22]. Chest compressions can raise ETCO_2_, while increasing ventilation volume or rate decrease ETCO_2_ [26]. A recent study modeled the ETCO_2_ percent decay with each ventilation in the absence of circulation, assuming constant volume and rate [27].

The aim of the present study was to investigate if the assessment of the evolution of ETCO_2_ during chest compression pauses could allow for ROSC detection. Our working hypothesis was: a decay in ETCO_2_ during a compression pause would indicate no ROSC while a constant or increasing ETCO_2_ would indicate ROSC. Furthermore, if the ECG shows an organized rhythm, ETCO_2_ trend could help to discriminate pulseless electrical activity (PEA) from a perfusing rhythm (PR).

## Methods

### Data collection

This is a retrospective study of adult out-of-hospital cardiac arrest (OHCA) episodes attended by Tualatin Valley Fire & Rescue (TVF&R), an ALS emergency medical service agency (Tigard, Oregon, USA) from 2006 through 2017. The database is part of the Portland Resuscitation Outcomes Consortium Epidemiological Cardiac Arrest Registry approved by the Oregon Health&Science University (OHSU) Institutional Review Board (IRB00001736). The database does not include patient identifying information.

Episodes were recorded using Heartstart MRx monitor-defibrillators (Philips, USA), equipped with capnography monitoring using sidestream technology (Microstream™, Oridion Systems Ltd., Israel) and CPR quality monitoring (Q-CPR™). Airway management techniques included bag-valve-mask (BVM) ventilation, supraglottic airway (SGA) devices and endotracheal tube (ETT). In the early years, CPR followed the 30:2 approach moving to continuous chest compressions (without pauses for ventilations) in 2012.

### Segment selection

Two biomedical engineers (JJG and JMR) visually inspected four signals extracted from the OHCA defibrillator recordings: capnogram, compression depth signal, ECG and transthoracic impedance (TTI). Only episodes, one per patient, with reliable capnograms were included in the study. Segments of capnography signal during pauses in chest compressions were identified in each episode. Only segments shorter than 20 s, with at least three ventilations, and with ETCO_2_ values equal or higher than 10 mmHg were included in the analysis. ETCO_2_ values lower than 10 mmHg were assumed to indicate absence of ROSC [5].

**Fig 1:**
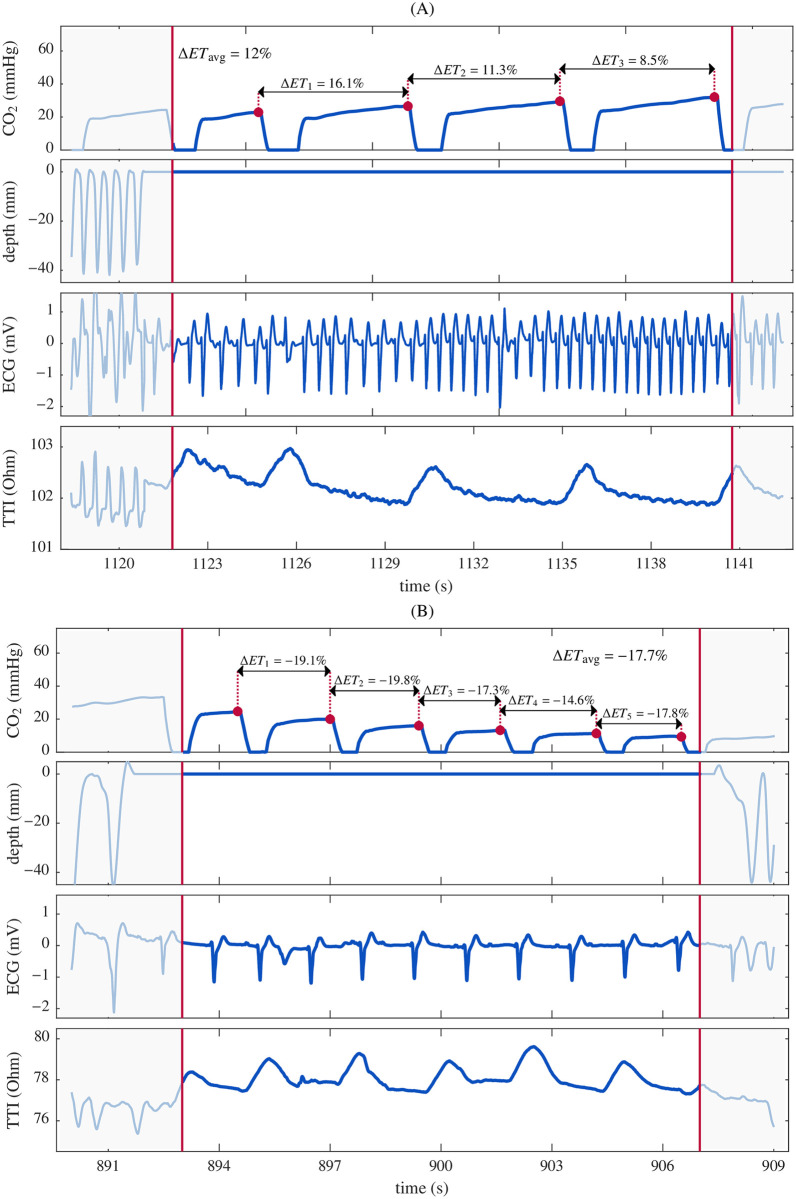
Examples of segment annotation. (A) ROSC segment and (B) non-ROSC (PEA) segment. Capnogram, compression depth, ECG and transthoracic impedance (TTI) signals are depicted for each segment.

The selected segments were then classified as ROSC or non-ROSC. We reviewed the ROSC annotations by the ALS providers and confirmed the presence of an organized rhythm in the ECG. Then, in case of doubt between an organized non-perfusing (PEA) or PR, we examined the TTI signal to locate its circulation component [28]. We selected a single ROSC segment per patient, the first segment without chest compressions after the first clinical annotation of ROSC. Conversely, several non-ROSC segments per single patient were included. Fig 1 shows two segments corresponding to ROSC (panel A) and non-ROSC (panel B). Capnogram, compression depth, ECG and TTI signals are depicted for each segment. In both segments, the compression depth signal is a flat line, reflecting the absence of chest compressions. The ECG in panel A corresponds to a PR. The presence of low amplitude ripples in the TTI between the large fluctuations caused by ventilation confirm circulation [28]. Conversely, the ECG in panel B corresponds to a PEA, confirmed by the absence of ripples in the TTI.

### Segments characterization

For each included segment, the following values were manually annotated in the capnogram (Fig 1): the start and end of the segment in seconds (*t*_s_, *t*_e_), depicted in the figure with vertical red lines; the number of ventilations within the segment (nv); and the ETCO_2_ value per ventilation (*ET*_*n*_), for *n* = 1…nv. *ET*_*n*_ were calculated as the maximum concentration of CO_2_ reached in the capnogram plateau, and are depicted in the figure with red dots. Once a segment met the inclusion criteria, it was characterized by four features:

- Mean ventilation rate (ventilations per minute, vpm): $v_{\text{r}}=\frac{\text{nv}}{t_{\text{e}}-t_{\text{s}}}\cdot 60$.
- *ET*_0_ (mmHg): the ETCO_2_ value for the first ventilation.
- Δ*ET*_*n*_ (%): percentage variation of *ET*_*n*_ between consecutive ventilations calculated as $\Delta ET_{\text{n}}=\frac{ET_{n}-ET_{n-1}}{ET_{n-1}}\cdot 100$ for *n* = 1…nv−1. Positive Δ*ET*_n_ means a positive trend in ETCO_2_ between consecutive ventilations while negative Δ*ET*_n_ means a negative trend. A zero value indicates a stable ETCO_2_.
- Δ*ET*_avg_ (%): the average variation calculated as the mean of percentage variation Δ*ET*_*n*_.

### Method for ROSC detection

Discrimination between ROSC and non-ROSC segments was conducted using the Δ*ET*_avg_ feature, as a metric of the positive or negative trend in ETCO_2_ variation. We compared the Δ*ET*_avg_ values with a detection threshold: segments presenting Δ*ET*_avg_ greater than the threshold were classified as ROSC. Values equal to or less than the threshold were classified as non-ROSC.

### Statistical analysis

Ten-fold cross-validation (multiple train/test split) was used to assess the predictive ability of the discrimination method. For each iteration, the discrimination threshold was set using the training set. The threshold was the value corresponding to the intersection between sensitivity (Se) and specificity (Sp). Patients were unrepeated in the training and test set for each iteration. No additional stratification was applied. Se was defined as the proportion of annotated ROSC segments that were detected as ROSC by our method. Sp was defined as the proportion of annotated non-ROSC segments that were detected as non-ROSC. Positive predictive values (PPV) and negative predictive values (NPV) were also calculated. Their corresponding 95% confidence intervals (CI) were reported.

Results were reported as median (IQR) since distributions did not pass the Lilliefors normality test. Comparison between groups was performed using the Wilcoxon rank sum test. *P*-values lower than 0.05 were considered significant. The distribution of *ET*_0_, *v*_r_ and Δ*ET*_avg_ per segment were depicted using box plots.

We also analyzed the results with respect to the airway management technique, ETT or SGA.

## Results

**Fig 2:**
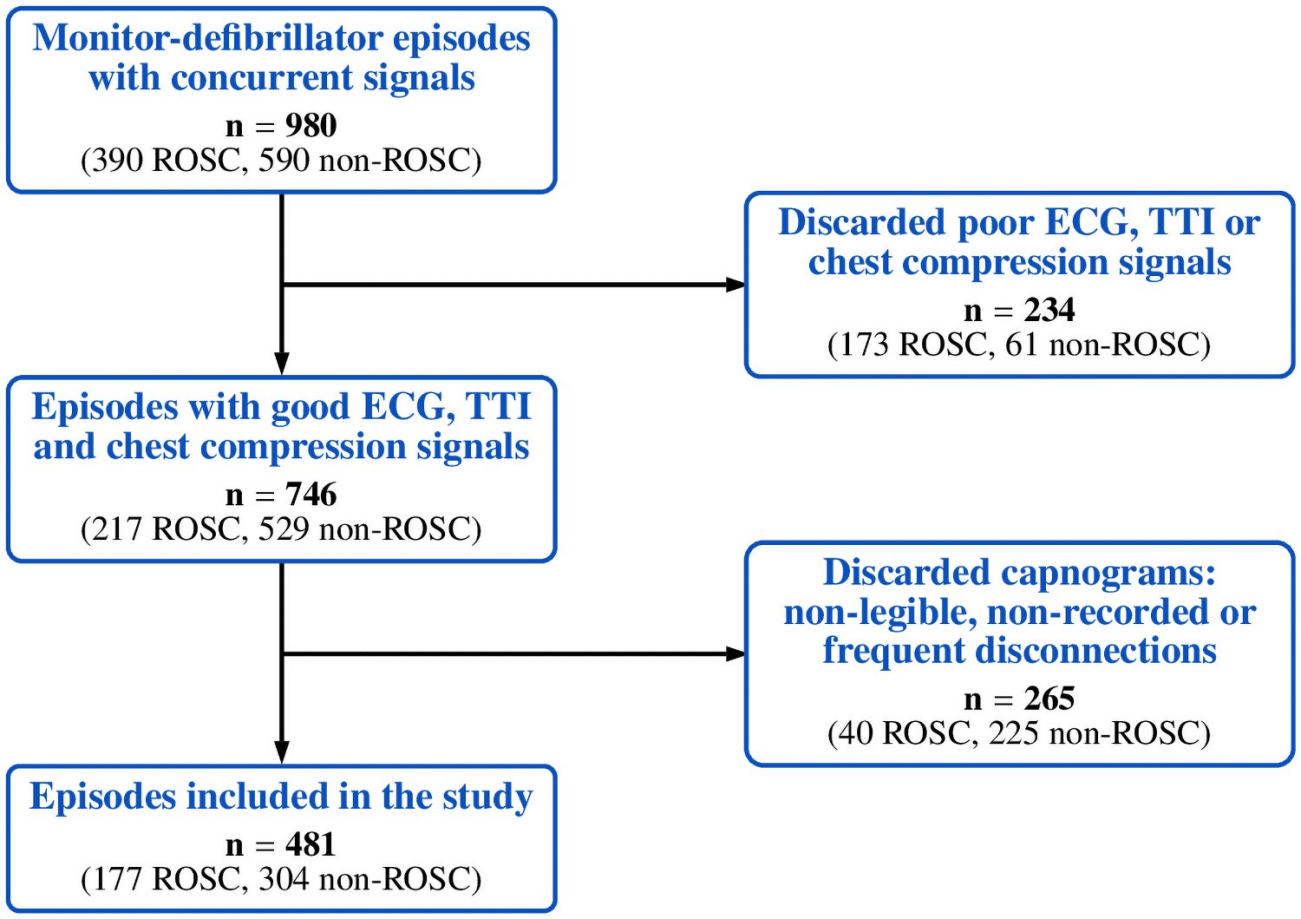
Flowchart of episode selection.

Concurrent signals of interest (capnogram, compression depth, ECG and TTI signals) were available in 980 adult OHCA episodes (one per patient), as illustrated in Fig 2, of which 390 included information regarding clinical ROSC annotation. Episodes with poor ECG, TTI or chest compression signal quality were discarded (173 ROSC; 61 non-ROSC). We also excluded episodes with high proportion of non-legible, non-recorded capnogram or capnogram presenting frequent disconnections (40 ROSC; 225 non-ROSC). A total of 481 (177 ROSC; 304 non-ROSC) OHCA episodes were eligible for analysis.

After applying the inclusion criteria for segment selection, 384 segments from 205 patients were included in the study. Median patient age was 66 years (55-79), and 63 of them (30.7%) were female. Eighty seven (87) patients had their airway managed with SGA (42.4%), and 115 with ETT (56.1%). Airway type was not known for the remaining 3 episodes (1.5%). Of the 384 segments, 130 were annotated as ROSC (33.9%) and 254 were annotated as non-ROSC (66.1%). Rhythm prevalence for non-ROSC segments was: 23 asystole, 193 PEA, 37 ventricular fibrillation, 1 ventricular tachycardia.

**Fig 3:**
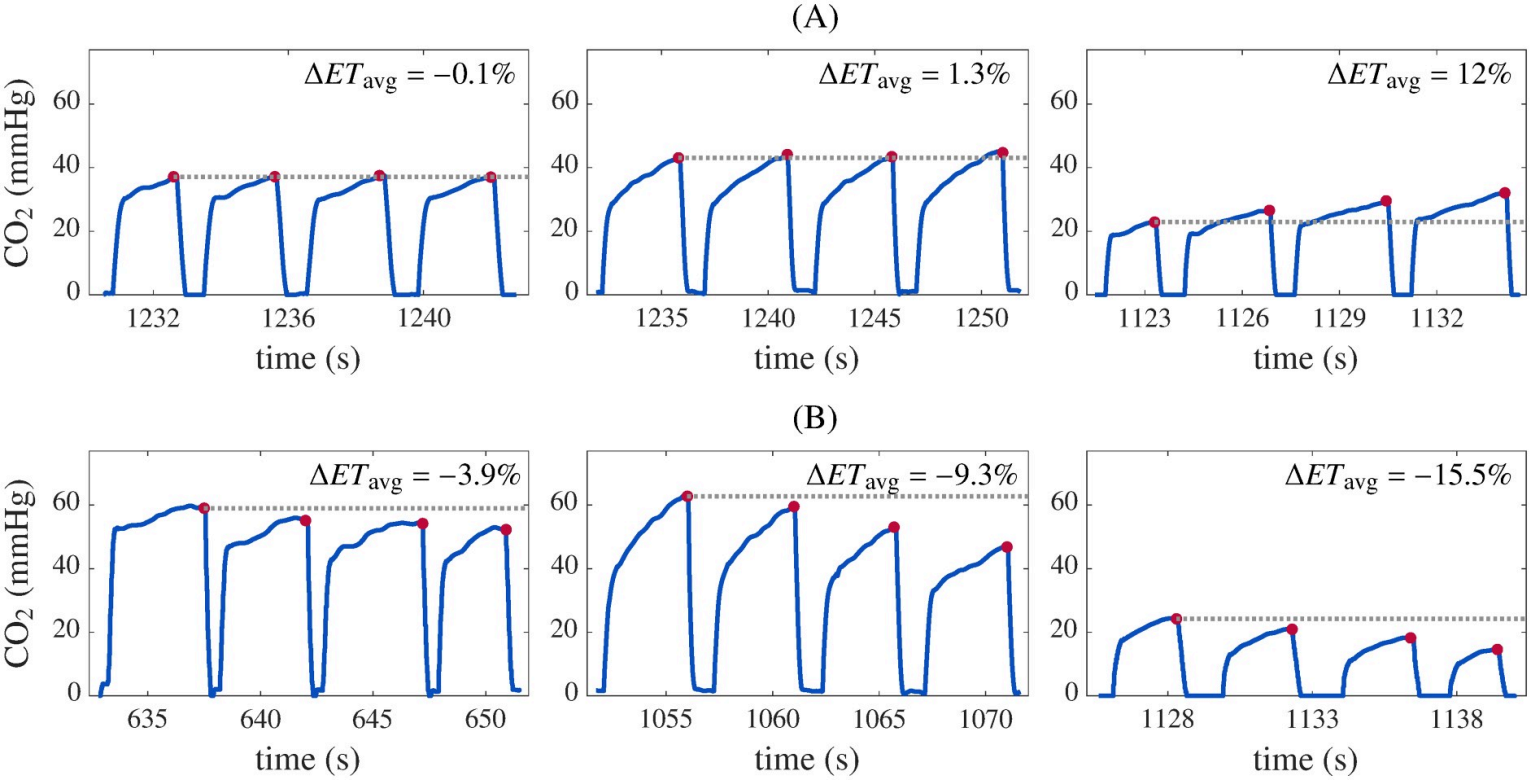
Illustration of the behavior of Δ*ET*_avg_. ROSC (A) and non-ROSC (B) capnogram segments with different Δ*ET*_avg_ values.

Fig 3 shows some examples of the calculation of the discrimination parameter Δ*ET*_avg_ for ROSC and non-ROSC segments.

Table 1 shows the distributions of the analyzed parameters. For the whole set, the median *v*_r_ was 13.3 (10.4, 17.5) vpm for ROSC segments, and 14.5 (11.1, 18.6) vpm for non-ROSC segments (*p* < 0.0001). *ET*_0_ was 45.8 (34.7, 58.4) mmHg for ROSC segments, and 29.4 (19.1, 39.6) mmHg for non-ROSC segments (*p* < 0.0001). Δ*ET*_avg_ presented values of 0.0 (-0.7, 0.9)% for ROSC segments, and -11.0 (-14.1, -8.0)% for non-ROSC segments (*p* < 0.0001).

**Table 1:** Segments characterization as a function of the airway type and ROSC condition. Values are reported as median (IQR).

	SGA		ETT		TOTAL	
	ROSC	non-ROSC	ROSC	non-ROSC	ROSC	non-ROSC
**Episodes**	48	39	80	35	130	75
**Segments**	48	115	80	132	130	254
**Duration (s)**	17.0 (16.0,18.6)	15.3 (12.4,18.2)	17.7 (16.2,19)	16.2 (13.0,18)	17.5 (16.1,18.7)	15.9 (12.6,18.0)
nv	4 (3,5)	3 (3,4)	4 (3,5)	3 (3,5)	4 (3,5)	3 (3,4)
*v*_**r**_ **(vpm)**	12.8 (11.0,17.2)	14.5 (12.3,17.8)^a^	13.6 (9.8,17.8)	14.6 (10.6,19.1)^b^	13.3 (10.4,17.5)	14.5 (11.1,18.6)^c^
***ET*_0_ (mmHg)**	50.9 (40.3,60.8)	30.3 (22.2,39.9)^c^	41.8 (32.0,55.8)	24.9 (15.1,37.7)^c^	45.8 (34.7,58.4)	29.4 (19.1,39.6)^c^
**Δ*ET*_avg_ (%)**	0.0 (-0.7,1.1)	-10.2 (-12.8,-7.3)^c^	0.3 (-0.5,1.3)	-11.4 (-14.5,-7.9)^c^	0.0 (-0.7,0.9)	-11.0 (-14.1,-8.0)^c^

nv: ventilations per segment; *v*_r_: ventilation rate in ventilations per minute (vpm); *ET*_0_: initial ETCO_2_; Δ*ET*_avg_: average ETCO_2_ variation.

^a^p = 0.012,

^b^p = 0.001,

^c^p<0.0001.

**Fig 4:**
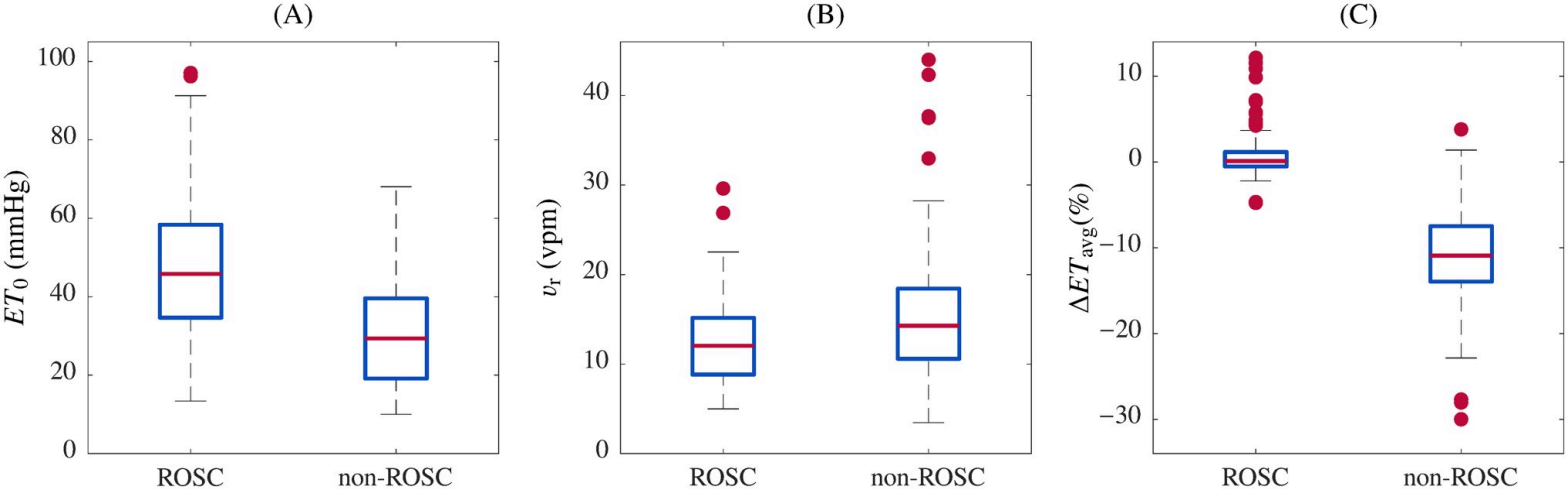
Statistical distributions for ROSC and non-ROSC segments. Initial ETCO_2_ (*ET*_0_), ventilation rate (*v*_r_), and average variation (Δ*ET*_avg_) for all ROSC and non-ROSC segments included in the study.

Fig 4 shows the distributions of *ET*_0_, *v*_r_ and Δ*ET*_avg_, for ROSC and non-ROSC segments. Distributions of *ET*_0_ and *v*_r_ presented wide overlapping ranges for ROSC and non-ROSC populations, while for Δ*ET*_avg_ a small overlap was observed.

Table 2 shows the ROSC/non-ROSC classification performance of our proposed classifier for the whole set and for the PEA/PR subset. Results are provided considering all ventilations in the segment, only the first three ventilations, and only the first two ventilations. When all the ventilations of each segment were considered, the median (IQR) duration was 16.3 (12.9, 18.1) s, Se was 95.4% and Sp was 94.9%. Considering the first 3 ventilations of each segment the duration was 11.9 (9.3, 14.8) s, Se was 93.8% and Sp 95.3%. Considering the first 2 ventilations of each segment the duration was 7.7 (6.0, 10.2) s, with Se 90.0% and Sp 89.4%. For PEA/PR, Se was 94.6% and Sp was 94.3% for all ventilations, 93.8% and 94.8% for the first 3 ventilations and 88.5% and 88.1% for the first 2 ventilations. The method confirmed the clinical decision of ROSC during the first chest compression pause in 95.4% of the episodes.

**Table 2:** ROSC detection results, as a function of the number of considered ventilations per segment, for the whole set and for the PEA/PR subset. Durations are reported as median (IQR). The 95% confidence intervals are in parenthesis.

Whole set	All ventilations	First 3 ventilations	First 2 ventilations
**Duration (s)**	16.3 (12.9, 18.1)	11.9 (9.3, 14.8)	7.7 (6.0, 10.2)
Se (%)	95.4 (90.1, 98.1)	93.8 (88.1, 97.0)	90.0 (83.5, 94.2)
Sp (%)	94.9 (91.4, 97.1)	95.3 (91.8, 97.4)	89.4 (84.9, 92.6)
PPV (%)	90.5 (84.3, 94.5)	91.0 (84.9, 94.9)	81.3 (74.0, 86.8)
NPV (%)	97.6 (94.7, 99.0)	96.8 (93.7, 98.5)	94.6 (90.9, 96.9)
**PEA/PR set**			
Se (%)	94.6 (89.1, 97.6)	93.8 (88.1, 97.0)	88.5 (81.7, 93.0)
Sp (%)	94.3 (90.0, 97.0)	94.8 (90.6, 97.3)	88.1 (82.7, 92.0)
PPV (%)	91.8 (85.8, 95.5)	92.4 (86.5, 96.0)	83.3 (76.2, 88.7)
NPV (%)	96.3 (92.4, 98.3)	95.8 (91.8, 98.0)	92.0 (87.0, 95.1)

Se: sensitivity (TP/(TP+FN)); Sp: specificity (TN/(TN+FP)); PPV: positive predictive value (TP/(TP+FP)); NPV: negative predictive value (TN/()TN+FN). TP: true positive; FP: false positive; TN: true negative; FN: false negative.

There were statistically significant differences between airway types for *ET*_0_ within the ROSC and non-ROSC populations (*p* = 0.01 in both cases). However, no significant differences were found for *v*_r_ (*p* = 0.87 and *p* = 0.97 for ROSC and non-ROSC, respectively) and for Δ*ET*_avg_ (*p* = 0.2 and *p* = 0.1, respectively). For ETT segments, Se and Sp were 91.2% (82.8%, 96.0%) and 92.4% (86.5%, 96.0%), respectively; whereas for SGA segments, Se and Sp were 95.8% (82.8%, 99.6%) and 96.5% (91.1%, 98.9%), respectively.

## Discussion

In this retrospective study, we sought to analyze the evolution of ETCO_2_ during chest compression pauses, that is, in the absence of blood flow generated by chest compressions, during OHCA interventions. We proposed a metric, the average ETCO_2_ variation across the ventilations within the pause, to perform as a threshold to discriminate ROSC from non-ROSC segments.

Several methods for the automated detection of circulation based on the ECG and the TTI signals can be found in the literature [28–34]. In general, they rely on the analysis of the signals during chest compression pauses, looking for an organized rhythm in the ECG and the circulation component of the TTI. Since ECG and TTI signals are typically the only available signals in automated external defibrillators, these methods were intended primarily for basic life support (BLS) settings.

During ALS, the capnogram can be useful for ROSC detection, as it is an indirect indicator of perfusion. Pokorná et al. studied the significance of a sudden increase in ETCO_2_ during ALS OHCA episodes [18]. ETCO_2_ values were higher when ROSC was achieved (*p* < 0.0001). Analyzing an increase of 10 mmHg in the measured ETCO_2_ during 2-min periods, they obtained a sensitivity of 80% with a specificity of 59%. Davis et al. analyzed the prediction of ROSC prior to a compression pause and non-ROSC indicators within the pause [35]. A heart rate greater than 40 bpm and a ETCO_2_ value above 20 mmHg pre-pause and a ETCO_2_ decay greater than or equal to 10 mmHg in less than 10 s, and ETCO_2_ < 20 mmHg intrapause, yielded a PPV of 95% and NPV of 99% in ROSC detection. Lui et al. evaluated the diagnostic accuracy of an abrupt and sustained increase in ETCO_2_ to indicate ROSC in OHCA patients [19]. For an ETCO_2_ rise greater than 10 mmHg, ROSC was detected with a sensitivity of 33% and a specificity of 97%. Brinkrolf et al. assessed the detection of ROSC by identifying ETCO_2_ trends in real time [16]. The study showed that ROSC time series presented larger percentages of positive trends than non-ROSC time series (*p* = 0.003). ROSC was detected with a sensitivity of 73.9% and a specificity of 58.4%. Finally, Elola et al. used the ECG and the TTI for ROSC assessment during chest compression pauses. When they included the mean ETCO_2_ value of the minute before the beginning of the compression pause in the classifier, they obtained high sensitivity and specificity values [36].

In this work, we demonstrated that the average percent variation of ETCO_2_ during chest compression pauses allows differentiation between non-ROSC and ROSC segments. The hypothesis behind was that, in non-ROSC scenarios, with no flow being generated by chest compressions, exhaled CO_2_ concentration is driven only by ventilations, and decays with each ventilation [27]. Conversely, ETCO_2_ would increase (or at least remain constant) as a result of the blood flow generated by spontaneous circulation.

This approach yielded a global sensitivity of 95.4% and specificity of 94.9% for predicting the presence of ROSC, and 94.6% and 94.3%, respectively, for the PEA/PR subset. Our approach did not rely on ETCO_2_ absolute measurements. In fact, the ETCO_2_ value measured in the first ventilation within the compression pause was not a sensitive indicator of ROSC in our database (see Fig 4).

ALS guidelines recommend not interrupting chest compressions for more than 10 s [5]. With our criterium of pauses shorter than 20 s we obtained good performance results, above 90% for all metrics. Our approach needed several ventilations to obtain measurement points in order to assess the ETCO2 evolution, but this may limit the applicability of the method. To test the algorithm with pauses closer to the recommended 10 s, we applied the method to the first three and two ventilations of the segments. In case of three ventilations, the median duration of the segments was 11 s. Performance was similar to the global results. In case of two ventilations, the median duration was 7.7 s and the performance slightly decreased, with values close to 90%, except for PPV (81.3%). Similar conclusions were derived from the analysis conducted with the PEA/PR subset.

During chest compression pauses for pulse check, the ECG signal is not affected by the artifact caused by compressions and it can be directly analyzed to establish whether the electrical activity of the heart is compatible with a pulse-generating rhythm. ECGs compatible with pulse-generating rhythms can be PEA (non-ROSC) or PR (ROSC). Direct observation of the evolution of ETCO_2_ in the capnogram would allow us to determine whether there is ROSC or not. In spite of the efforts made, to the best of our knowledge there is no monitor-defibrillator providing an automated assessment for the detection of ROSC [23]. Our method provides a valuable metric from waveform capnography and could be considered as another step towards an automated algorithm for confirming circulation. Nevertheless, including additional parameters such as initial ETCO_2_, ventilation rate or ECG features could yield better results, especially for PEA/PR discrimination. The reliable and automated detection of ROSC based on the signals and data provided by the monitor-defibrillator would be valuable, on the one hand, to prevent prolonged detrimental interruptions of CPR with PEA, and, on the other hand, to avoid potentially harmful chest compressions and unnecessary drug administration to patients.

### Limitations

A limitation of our work comes from the need to obtain measurement points for several ventilations during pauses in compressions. This resulted in the inclusion of pauses longer than recommended or with ventilation rates differing from recommendation, which should not be frequent in the practice. The goal for ventilation rate is normally assessed on a minute-by-minute basis or for the whole episode. However, ventilation rate may be locally either lower or higher than the average. Considering segments with ventilation rates close to 10 vpm implied discarding the majority of potential segments, most of them for having much higher rates. Aware of this limitation, we analyzed the first two ventilation segments (see Table 2). As expected, results were worse but reflected the goodness of the method when in adherence to the guidelines.

Our study did not consider the variability in ventilation volumes (unavailable in our database and generally in the field). We hypothesize that our method would be a reliable tool in clinical practice when ventilations are administered with stable volumes.

In our study, ROSC was confirmed by clinicians’ annotations and by the analysis of the ECG and of the circulation component in the TTI. This methodology has been widely used in the literature as a surrogate for circulation assessment, and constitutes the best possible approach in the absence of invasive indicators. Still, we recognize the challenge of a real determination of ROSC in pre-hospital settings. Thus, we only selected the first ROSC segment for each episode. This restrictive criterium may, however, limit the applicability of the method.

The database was completed over a long period of time. Guidelines changed and the EMS systems moved from 30:2 compression-ventilation ratio to continuous compressions. Recommended depth and rate of chest compressions also changed (2010 and 2015 guidelines). However, the results of this study should not be affected as the analysis was performed during chest compression pauses.

All the recordings of the database were obtained using a single capnograph manufacturer ETCO_2_ levels could be slightly influenced by the equipment, so it would be helpful to validate our findings using capnographs from different manufacturers. Also, a large number of recordings were discarded due to poor signal quality. This work would deserve validation with more data to ensure that our results are generalizable.

## Conclusion

Assessment of ETCO_2_ variation during pauses in chest compressions is a valuable metric to detect ROSC. A negative value would suggest non-ROSC while a constant or positive value would reflect ROSC. The metric we propose could help confirm the presence or absence of circulation during compression pauses, for ALS agencies that have ETCO_2_ monitoring capability. However, performance slightly worsened for pauses less than 10 s, so further research is necessary to obtain a feasible ROSC indicator from waveform capnography.

## Supporting information

S1 File Segment characterization. Detailed results per recording, segment and individual ventilation within each segment (see Table 1 for reference).
(XLSX)
